# Identification of key genes in calcific aortic valve disease via weighted gene co-expression network analysis

**DOI:** 10.1186/s12920-021-00989-w

**Published:** 2021-05-21

**Authors:** Jin-Yu Sun, Yang Hua, Hui Shen, Qiang Qu, Jun-Yan Kan, Xiang-Qing Kong, Wei Sun, Yue-Yun Shen

**Affiliations:** 1 Department of Cardiology, The First Affiliated Hospital of Nanjing Medical University, Nanjing, 210000 China; 2 Department of Cardiology, Liyang People’s Hospital, Liyang, 213300 China

**Keywords:** Calcific aortic valve disease, Weighted gene co-expression network analysis, Differentially expressed genes, Integrated bioinformatics analysis

## Abstract

**Background:**

Calcific aortic valve disease (CAVD) is the most common subclass of valve heart disease in the elderly population and a primary cause of aortic valve stenosis. However, the underlying mechanisms remain unclear.

**Methods:**

The gene expression profiles of GSE83453, GSE51472, and GSE12644 were analyzed by ‘limma’ and ‘weighted gene co-expression network analysis (WGCNA)’ package in R to identify differentially expressed genes (DEGs) and key modules associated with CAVD, respectively. Then, enrichment analysis was performed based on Gene ontology (GO) and Kyoto Encyclopedia of Genes and Genomes (KEGG) pathway, DisGeNET, and TRRUST database. Protein–protein interaction network was constructed using the overlapped genes of DEGs and key modules, and we identified the top 5 hub genes by mixed character calculation.

**Results:**

We identified the blue and yellow modules as the key modules. Enrichment analysis showed that leukocyte migration, extracellular matrix, and extracellular matrix structural constituent were significantly enriched. *SPP1*, *TNC*, *SCG2*, *FAM20A*, and *CD52* were identified as hub genes, and their expression levels in calcified or normal aortic valve samples were illustrated, respectively.

**Conclusions:**

This study suggested that *SPP1*, *TNC*, *SCG2*, *FAM20A*, and *CD52* might be hub genes associated with CAVD. Further studies are required to elucidate the underlying mechanisms and provide potential therapeutic targets.

**Supplementary Information:**

The online version contains supplementary material available at 10.1186/s12920-021-00989-w.

## Introduction

Calcific aortic valve disease (CAVD) is the most common subclass of valve heart disease in the elderly population and a primary cause of aortic valve stenosis [1]. It was reported that the incidence of aortic valve stenosis was 2% in patients ≥ 65 years old, whereas aortic valve sclerosis occurred in 26% of these patients [2, 3]. Considering the prolonged life expectancy and aging of the population, the burden of CAVD is expected to substantially increase from 2.5 million in 2000 to 4.5 million in 2030 [4], thus conferring a high economic and health burden worldwide [5, 6].

In CAVD, the fibro-calcific remodeling and pathological thickening of the aortic valve disturb pressure overload and hemodynamic stability. The following progressive cardiac outflow obstruction and left ventricular hypertrophy are likely to result in heart failure and even premature death within a few years [4, 7]. Accumulating studies have revealed that CAVD is a complex multistage disease with sequential and interacting processes, including endothelial dysfunction/injury, lipid deposition, inflammation, extracellular matrix remodeling, dystrophic calcification, and so forth [8–10]. However, mechanisms underlying the development or progression of CAVD remain unclear, and there lacks conservative treatment against CAVD. Surgical or transcatheter aortic valve replacement is the only available treatment, while pharmacotherapy beyond valve replacement is still limited [5, 11].

Weighted gene co-expression network analysis (WGCNA) is a bioinformatics algorithm [12], which has been widely applied to explore the changes of transcriptome expression patterns in complex diseases and to identify gene modules associated with clinical features [13–15]. Compared with the standardized analysis of differentially expressed genes (DEGs), WGCNA is a powerful systematic analysis method to recognize the higher-order correlation between genes instead of detecting disease-related individual genes. Our study identified DEGs between calcified and normal aortic valve samples based on the gene expression profiles of GSE83453 and GSE51472, GSE12644. Moreover, WGCNA was performed on the GSE83453 dataset to screen key genes and modules related to CAVD. Then, the enrichment analysis of genes in key modules was used to explore the molecular mechanisms underlying CAVD. Finally, we identified hub genes related to CAVD and establish a protein–protein interaction (PPI) network.

## Materials and methods

### Microarray data

**Fig. 1:**
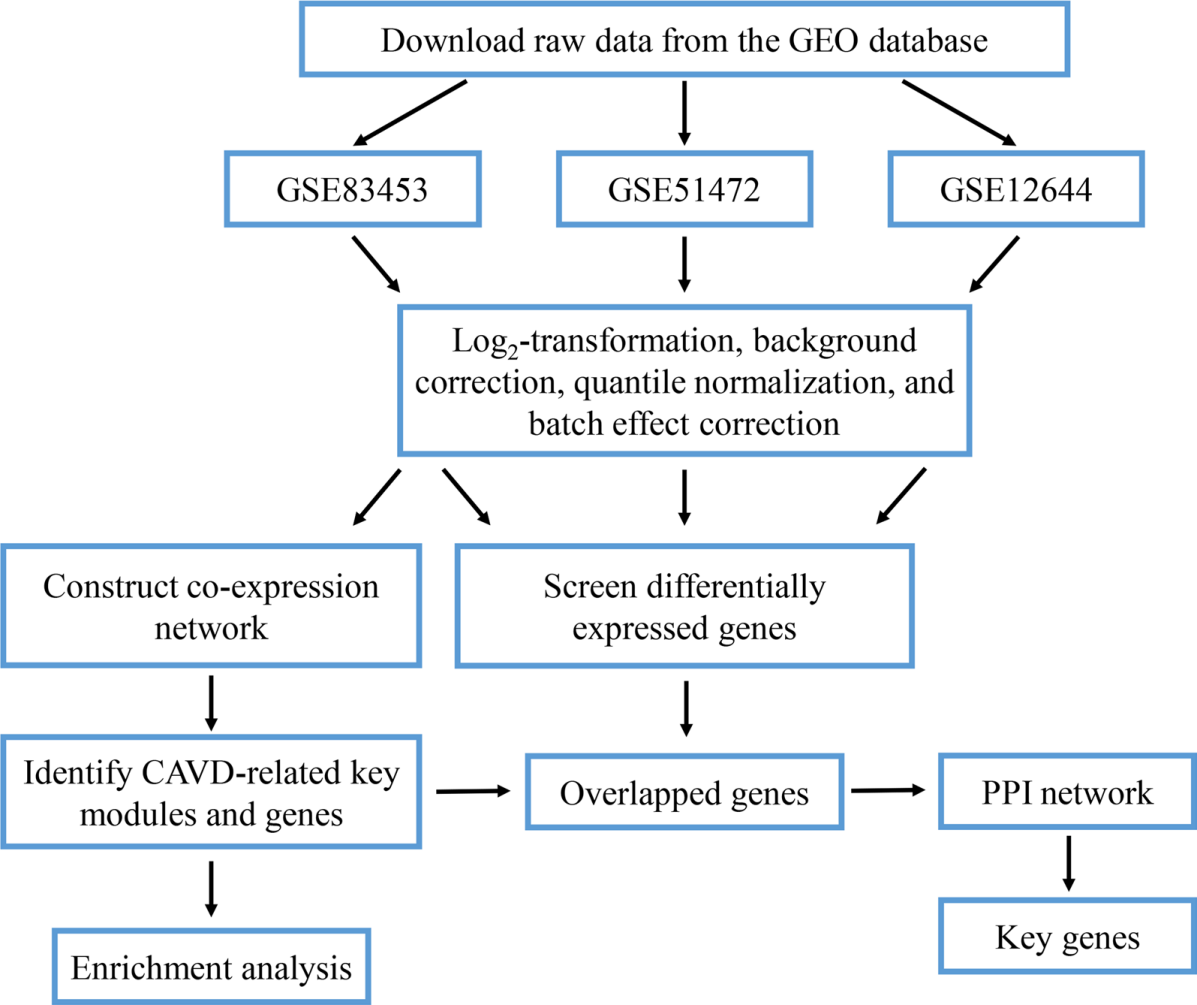
Flow chart of data preparation, processing, and analysis. *GEO* Gene Expression Omnibus, *CAVD* calcific aortic valve disease, *PPI* protein–protein interaction

The gene expression profiles of GSE83453 [16], GSE51472 [17], and GSE12644 [18] were acquired from the Gene Expression Omnibus database. All three gene expression profiles were acquired from human samples. Dataset GSE83453 was performed based on the platform GPL10558 (Illumina HumanHT-12 V4.0 expression beadchip), which includes 9 calcified aortic valve samples and 8 normal aortic valve samples [16]. Series GSE51472 was performed by GPL570 (Affymetrix Human Genome U133 Plus 2.0 Array) and included 5 calcified aortic valve samples and 5 normal aortic valve samples [17]. GSE12644, based on the GPL570 platform, included 10 calcified and 10 normal aortic valve samples (Fig. 1).

### Data pre-processing and DEG screening

We performed log_2_-transformation, background correction, and quantile normalization on the expression profiles of GSE83453, GSE51472, and GSE12644 using the ‘linear models for microarray data (limma)’ package in R 3.6.1 software (R Foundation for Statistical Computing, Vienna, Austria). The probe IDs were converted into gene symbols according to the annotation file. For multiple probes mapping to a single gene, the average expression value of all its corresponding probes was used. Importantly, the batch effect was corrected using the combat function of the ‘SVA’ package, which is a widely used empirical Bayes method for batch correction [19]. To control the false discovery rate caused by multiple testing, the adjusted *P*-value was applied. After pre-processing, DEGs between calcified and normal aortic valve samples were screened with a threshold of adjusted *P*-value < 0.05 and |log_2_ fold‐change (FC)| ≥ 0.5 by ‘limma’ package. Furthermore, the DEGs were visualized as a volcano plot and heatmap using ‘ggplot2’ and ‘pheatmap’ package.

### Weighted gene co‐expression network analysis (WGCNA)

WGCNA is an algorithm for constructing a co-expression network, which reveals the correlation patterns across genes and provides the biologically functional interpretations of network modules. As previously described [20], we selected the top 25% most variant genes in GSE83453 to construct a co-expression network using the ‘WGCNA’ package (version 1.60). After evaluating the presence of obvious outliers by cluster analysis, the one-step network construction function was used to construct the co-expression network and identify key modules. Moreover, to identify the significance of each module, we summarized the module eigengene (ME) based on the first principal component of the module expression, and the module-trait relationships were assessed according to the correlation between MEs and clinical traits. Then, we evaluated the correlation strength by module significance (MS), referring to the average absolute gene significance (GS) of all genes within one module. Notably, the GS value was determined by the log_10_-transformation of the *P*-value in the linear regression between expression and clinical traits. In general, the modules with the highest MS values were considered as the key modules.

Furthermore, we used the modulePreservation function to evaluate the preservation levels of key modules. Z_summary_ analysis combines different preservation statistics into one single overall measure of preservation. According to WGCNA instruction, a higher Z_summary_ value indicated the stronger the evidence that a module should be preserved: the module with Z_summary_ value < 2 indicated ‘no evidence’, 2 < Z_summary_ value < 10 indicated ‘weak evidence’, and Z_summary_ value > 10 indicated strong evidence. Additionally, to confirm the clustering ability of the key modules, we also performed principal component analysis (PCA) on the gene expression profile of the key modules.

### Enrichment analysis of genes in key modules

To understand the biological meaning of the genes in key modules, we used the ‘clusterProfiler’ package to perform Gene Ontology (GO) and Kyoto Encyclopedia of Genes and Genomes (KEGG) pathway enrichment analysis. Moreover, pathway and process enrichment analysis were also conducted and visualized by Metascape [21], a biologist-oriented tool for analyzing systems-level datasets. To illustrate the relationships between the terms, a subset of terms was further selected and rendered as a network plot (with a similarity of > 0.3). Each node represented an enriched term and colored first by its cluster ID. Furthermore, we performed association and enrichment analysis using DisGeNET [22] and TRRUST [23] database and visualized by ‘ggplot2’ package. DisGeNET is a database of gene-disease associations, which collects one of the largest publicly available collections of genes and human diseases-related variants [22]. TRRUST is a manually-curated database of transcriptional regulatory networks based on a sentence-based text mining approach, containing 8444 and 6552 transcription factor-target regulatory relationships of 800 human and 828 mouse transcription factors, respectively [23].

### PPI network construction and identification of hub genes

In WGCNA, we defined the genes from key modules with |module membership (MM)|≥ 0.8 and |GS|≥ 0.2 as key genes. After overlapping the DEGs and key genes from WGCNA, we inputted these genes into the STRING (http://string-db.org) database to collect the interaction of target proteins with a medium confidence score of > 0.4 [24] and constructed a PPI network by Cytoscape software (v3.7.2) [25]. Further, we identified the top 5 hub genes using the Cytoscape plug-in software ‘cytoHubba’ based on mixed character calculation.

### The expression of hub genes in calcified or normal aortic valve sample

We compared the expression of hub genes between calcified or normal aortic valves from GSE83453 by Student’s t-test. The ‘ggplot2’ and ‘ggsignif’ packages were used to create the box plots of the expression of hub genes.

## Results

### DEGs between calcified and normal aortic valve

**Fig. 2:**
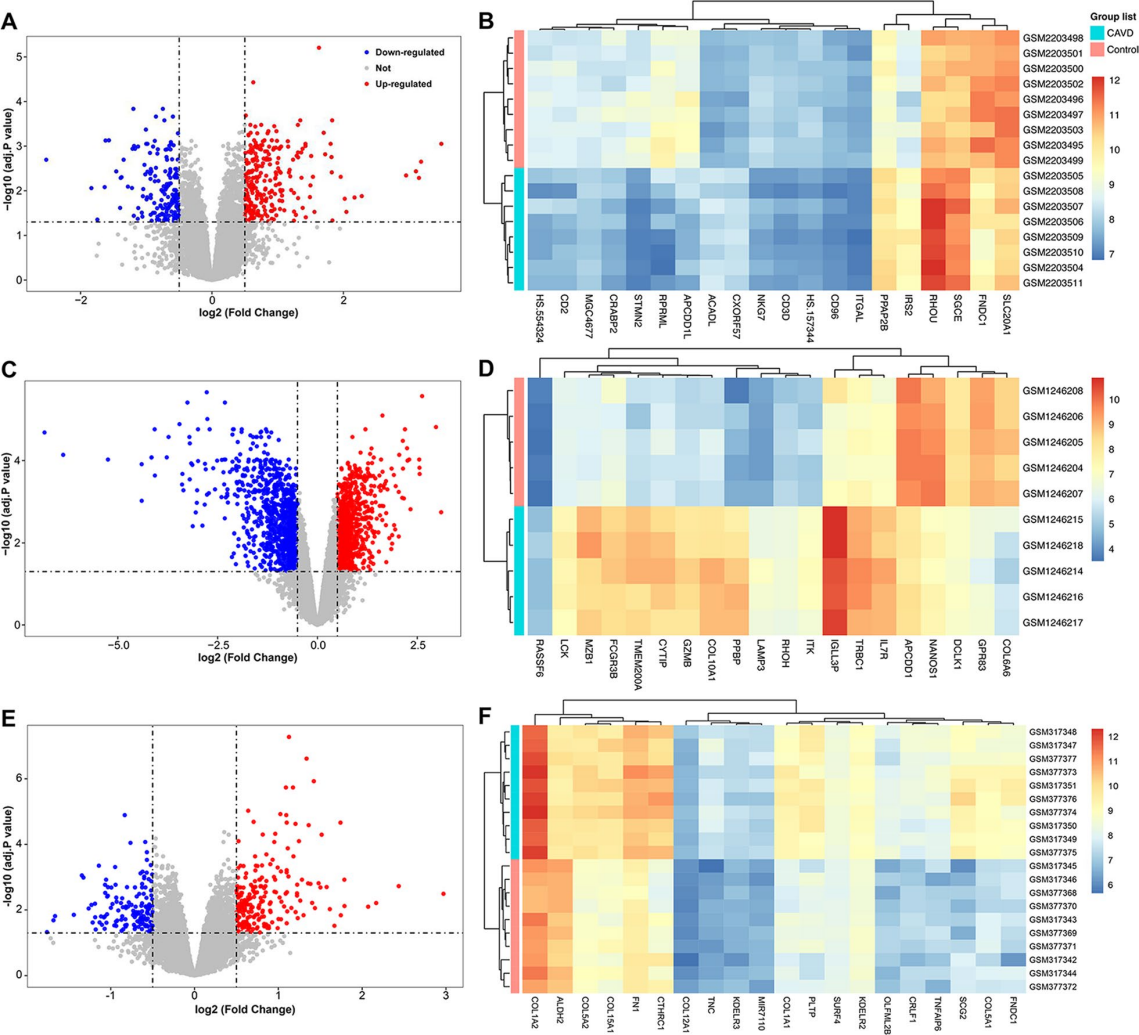
Identification of differentially expressed genes between calcified and normal aortic valve samples. Volcano plots of the differential gene expression data from **a** GSE83453, **c** GSE51472, and **e** GSE12644. In the volcano plots, the red points show up-regulated genes (log_2_FC ≥ 0.5 and adjusted *P*-value < 0.05), whereas the blue points represent down-regulated genes. Heatmap of the top 10 differentially expressed genes based on **b** GSE83453, **d** GSE51472, and **f** GSE12644. The color intensity (from red to blue) suggests the higher to lower expression. *FC* fold‐change

After data pre-processing of the dataset GSE83453, GSE51472, and GSE1264, we screened DEGs according to the cut-off criterion of adjusted *P*-value < 0.05 and |log_2_ FC| ≥ 0.5. In GSE83453, we identified 269 up-regulated and 170 down-regulated genes. The volcanic diagram for all genes and the expression heatmap of the top 10 DEGs are shown in Fig. 2a, b. In GSE51472, 963 genes were up-regulated, and 978 genes were down-regulated (Fig. 2c, d). A total of 28 up-regulated and 11 down-regulated genes were identified in GSE12644 (Fig. 2e, f).

### Construction of co‑expression network and key modules identification

**Fig. 3:**
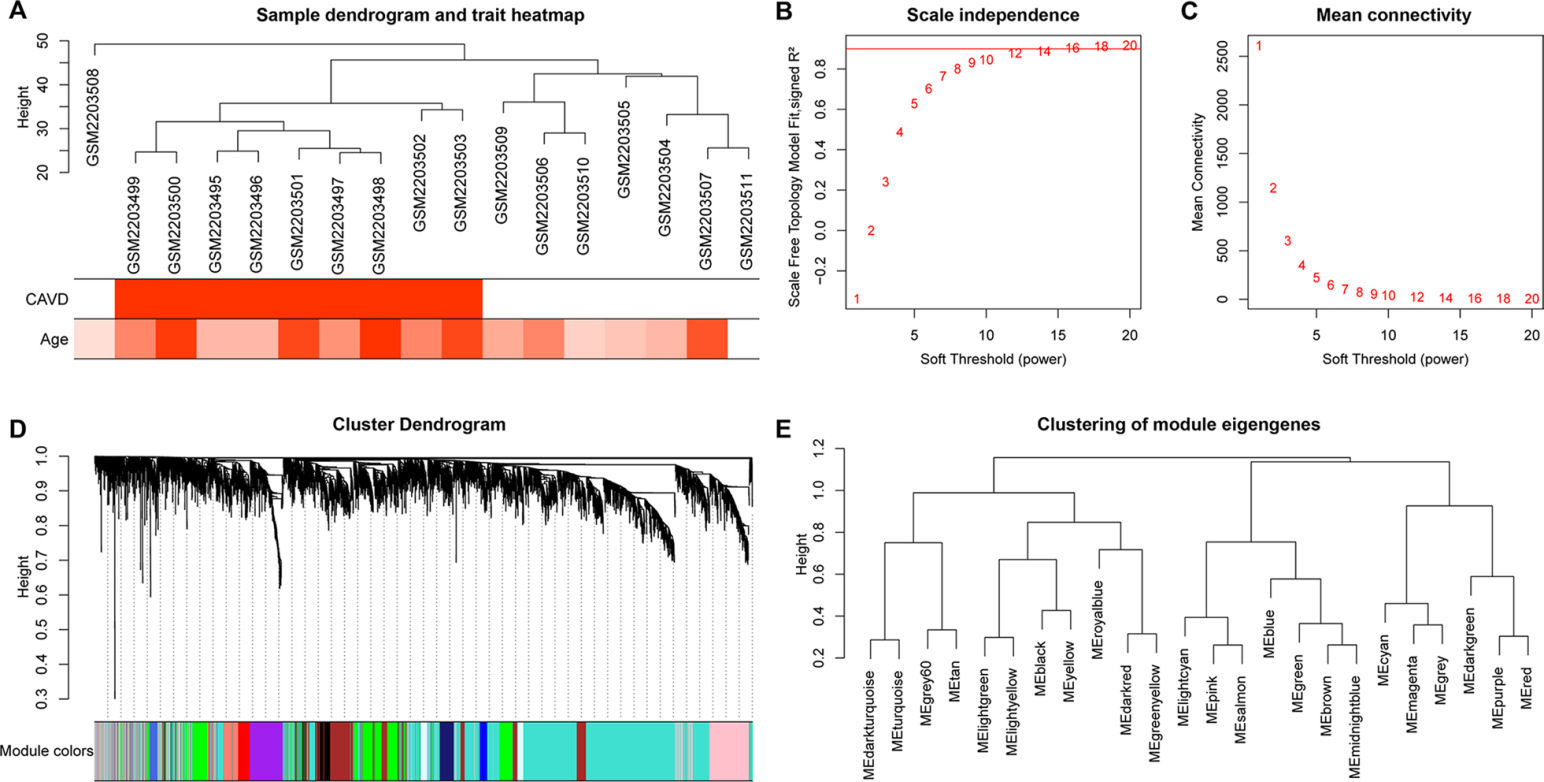
Sample clustering and network construction of the weighted co-expressed genes. **a** Clustering dendrogram of 9 calcified and 8 normal aortic valve samples. The color intensity was proportional to disease status (calcified or normal aortic valve) or age. Analysis of **b** the scale-free fit index and **c** the mean connectivity for various soft-thresholding powers. The soft-thresholding power of 12 was selected based on the scale‑free topology criterion. **d** Dendrogram clustered based on a dissimilarity measure (1-TOM). Gene expression similarity is assessed by a pair-wise weighted correlation metric and clustered based on a topological overlap metric into modules. Each color below represents one co-expression module, and every branch stands for one gene. **e** The cluster dendrogram of module eigengenes

With a scale-free network and topological overlaps, a hierarchical clustering tree was created based on the dynamic hybrid cut (Fig. 3a). Based on the scale‑free topology criterion, the soft-thresholding power of 12 was selected (scale-free R^2^ = 0.88; Fig. 3b, c), and a total of 24 modules were identified for further analysis. In addition, the gray module included all genes that could not be put into any other modules. The cluster dendrogram of the modules is shown in Fig. 3d, whereas the clustering of module eigengenes is provided in Fig. 3e.

**Fig. 4:**
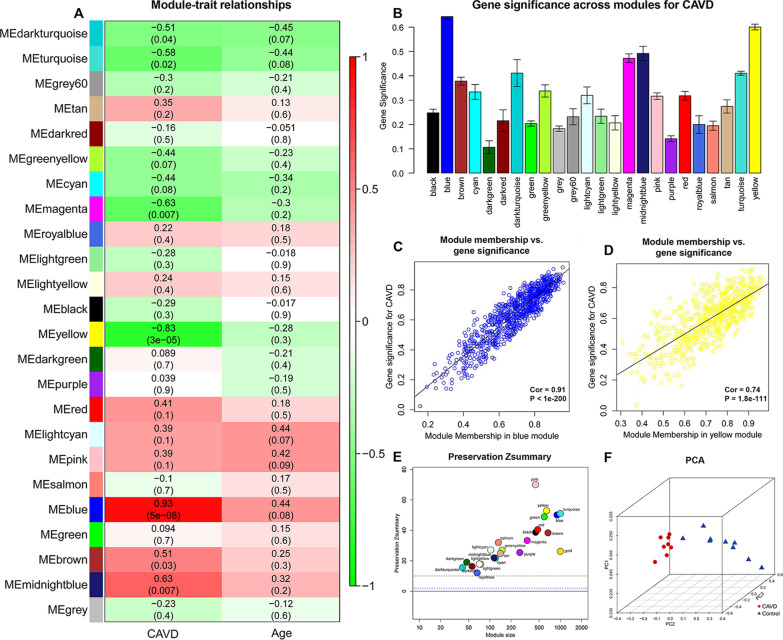
The identification of key modules via weighted gene co-expression network analysis. **a** Heatmap of the correlation between module eigengenes and the disease status of CAVD. The corresponding correlation coefficient along with *P*-value is given in each cell, and each cell is color-coded by correlation according to the color (legend at right). **b** Barplot of mean gene significance across modules associated with CAVD. A higher mean gene significance indicates that the module is more significantly related to CAVD. The blue and yellow modules were the most significantly correlated with CAVD. Scatter plot of module eigengenes in the **c** blue module and **d** yellow module. **e** Module preservation analysis based on Z_summary_. Each point represents a module, and the dashed blue and green lines indicate the threshold of 2 and 10, respectively. A module with a Z_summary_ of < 5 would be considered non-preserved. **f** The principal component analysis of the genes in blue and yellow modules. *CAVD* calcific aortic valve disease, *PCA* principal component analysis

Moreover, we analyzed the association of gene modules with CAVD status and age (Fig. 4a). The blue module showed the highest positive correlation (r = 0.93, *P* = 5e-08), while the yellow module was the most negatively associated with CAVD (r = -0.83, *P* = 3e-05). As shown in Fig. 4b, the blue and yellow modules were also the two most significantly correlated with CAVD. Therefore, we identified the blue and yellow modules as key modules for further analysis. A total of 436 and 82 genes were included in the blue and yellow modules, respectively. Additionally, we illustrated the correlation between module membership and GS for CAVD in blue (correlation coefficient = 0.91, P < 1e−200) and yellow module (correlation coefficient = 0.74, P = 1.8e−111), respectively (Fig. 4c, d).

Figure 4e shows the module preservation statistics, and the Z_summary_ values of both blue and yellow modules were > 10. The PCA on the genes in blue and yellow modules illustrated the overlap of samples within the CVAD or control groups, which suggested the high-level clustering ability of the key modules (Fig. 4f).

### Enrichment analysis of key modules

**Fig. 5:**
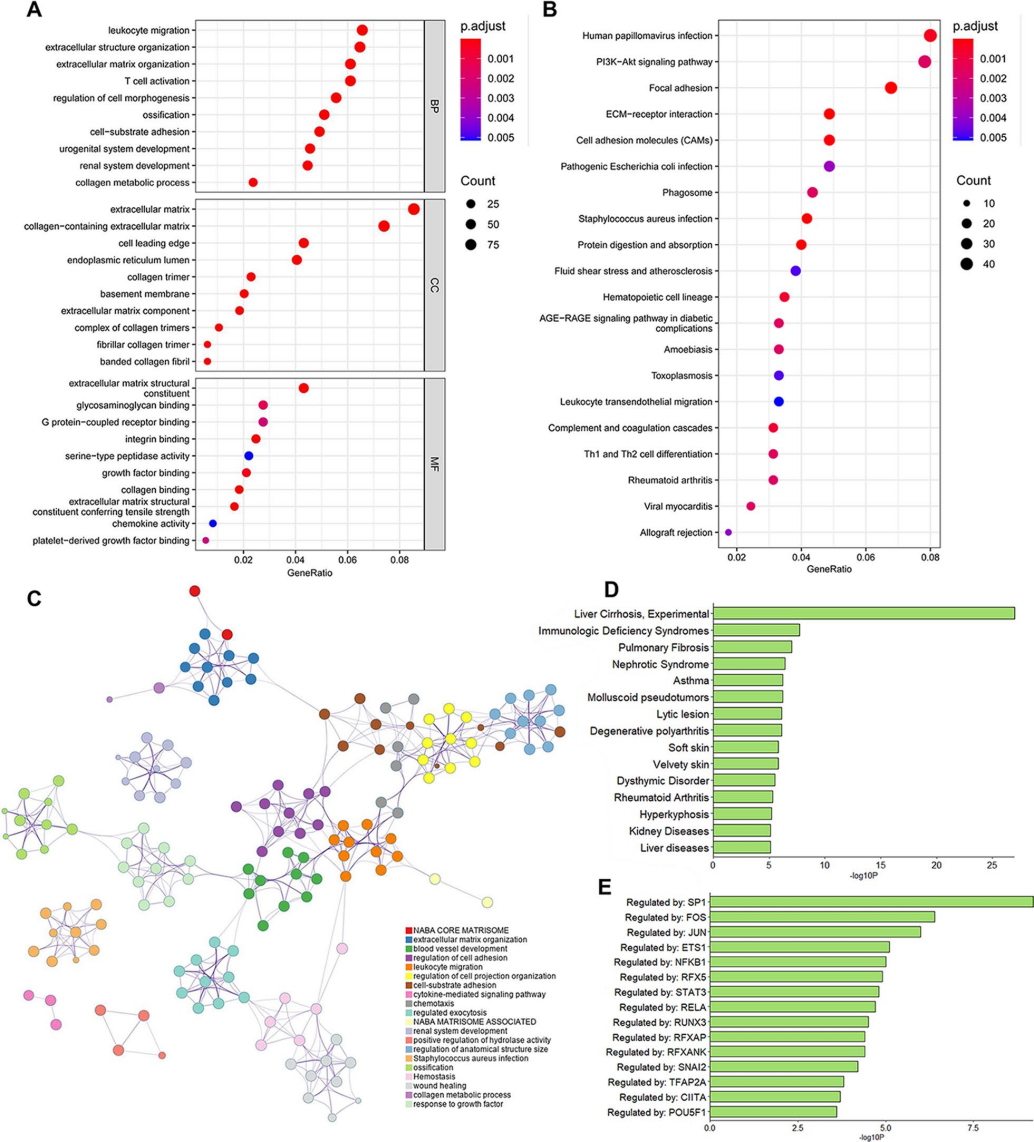
Enrichment analysis of key modules. **a** Gene ontology and **b** Kyoto Encyclopedia of Genes and Genomes pathway enrichment analysis in blue and yellow modules. The significance of enrichment gradually increases from blue to red, and the size of the dots indicates the number of genes contained in the corresponding pathway. **c** The network of enriched terms. Each node represents an enriched term and is colored by cluster ID. Nodes sharing the same cluster ID are typically close to each other. **d** Summary of enrichment analysis in DisGeNET. **e** Summary of enrichment analysis in TRRUST

We performed functional enrichment analysis on the blue and yellow modules based on GO and KEGG databases. As shown in Fig. 5a, the ontology encompasses three domains (biological process, cellular component, and molecular function). The enriched biological processes were mainly involved in leukocyte migration, extracellular structure organization, extracellular matrix organization, T cell activation, cell-substrate adhesion, and renal system development. The cellular components were mainly enriched in the extracellular matrix and collagen-containing extracellular matrix, whereas the enriched molecular functions were mainly involved in extracellular matrix structural constituent. In Fig. 5b, KEGG pathway analysis shows that the human papillomavirus infection and PI3K-Akt signaling pathway were the most enriched pathways, followed by focal adhesion, extracellular matrix-receptor interaction, cell adhesion molecules, and pathogenic *Escherichia coli* infection. In addition, Fig. 5c shows the top 20 clusters of the pathway and process enrichment analysis, such as regulation of cell adhesion, regulation of cell projection organization, ossification, collagen metabolic process, and so forth.

Moreover, the enrichment analysis in DisGeNET showed that the genes in key modules were significantly associated with liver cirrhosis, immunologic deficiency syndromes, and pulmonary fibrosis (Fig. 5d). Based on the TRRUST database, we also identified multiple transcription factors associated with the genes in key modules, including SP1, FOS, JUN, ETS1, NFKB1, and RFX5 (Fig. 5e).

### PPI network construction and identification of hub genes

**Fig. 6:**
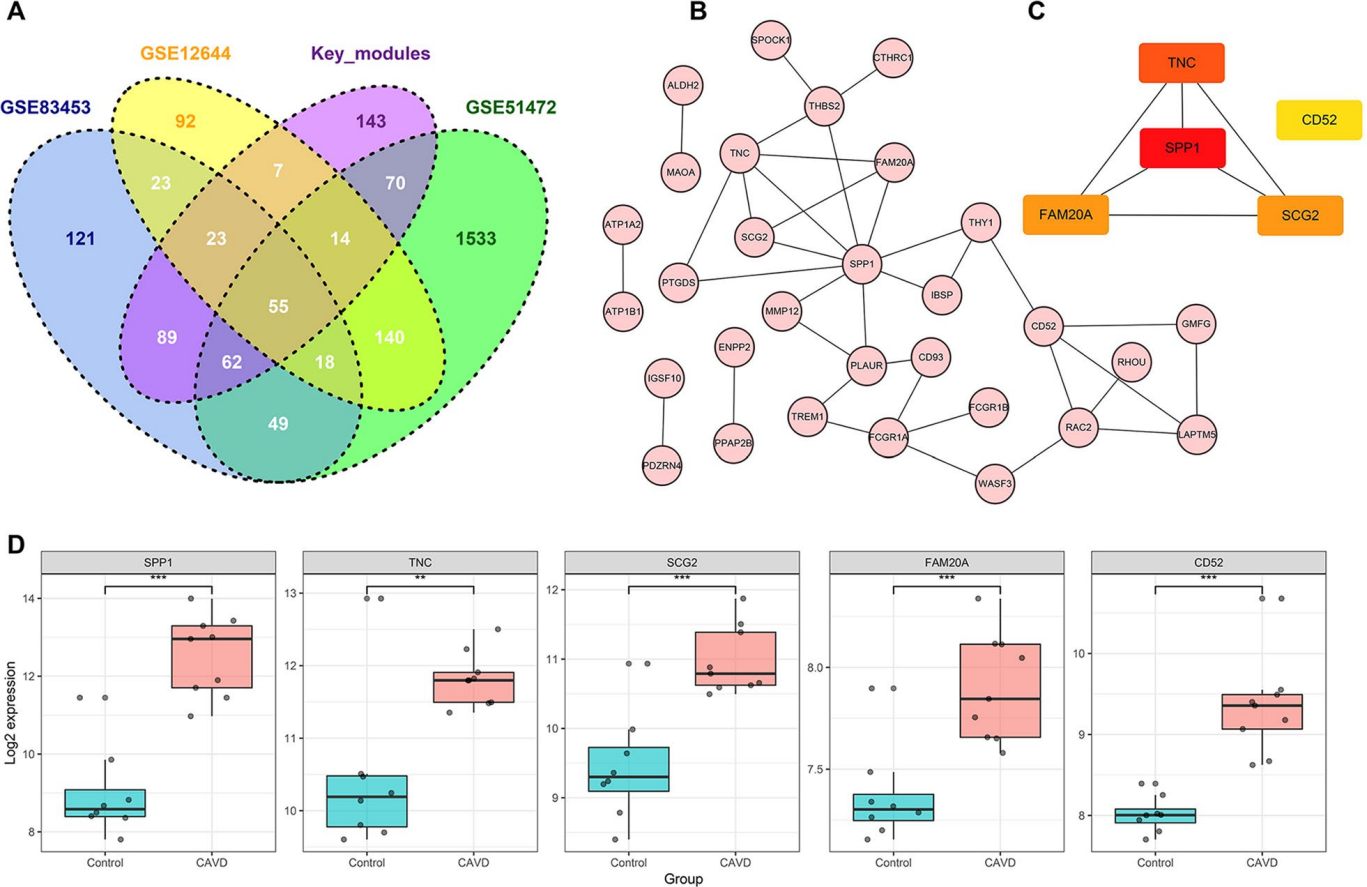
PPI network construction and identification of hub genes. **a** The Venn diagram of key genes from the key modules and DEGs from GSE83453, GSE51472, and GSE12644. **b** The protein–protein interaction network of the overlapped genes. **c** 5 hub genes identified by ‘cytoHubba’ according to mixed character calculation. The significance of hub genes gradually increases from yellow to red. **d** The expression levels of hub genes in calcified or normal aortic valve samples from GSE83453. Boxplots show the median, 25–75% percentiles, and range of log_2_ expression value. *P < 0.05; **P < 0.01; ***P < 0.001 (Student’s t-test)

With a threshold of |MM|≥ 0.8 and |GS|≥ 0.2, 252 and 210 key genes were identified from the blue and yellow modules, respectively. After overlapping the DEGs and key genes, a total of 55 genes were screened out (Fig. 6a; Additional file 1: Table 1). The PPI network of these genes is shown in Fig. 6b. Moreover, CytoHubba revealed the top 5 hub genes according to the topological parameters of the interaction network, including secreted phosphoprotein 1 (*SPP1*), tenascin C (*TNC*), secretogranin II (*SCG2*), family with sequence similarity 20-member A (*FAM20A)*, and *CD52* (Fig. 6c). As shown in Fig. 6d, the expression of *SPP1*, *TNC*, *SCG2*, *FAM20A*, and *CD52* was all significantly up-regulated in calcific aortic valve disease.

## Discussion

Compared with previous studies[26, 27], we used three gene expression profiles from 24 human calcified aortic valve samples and 23 normal samples, and both expression analysis and weighted co-expression network analysis were applied to identify key genes related to CAVD comprehensively. Our study performed WGCNA to reveal the correlation patterns between genes across samples and interpret the straightforward biological function of gene network modules. We also used multiple databases in the enrichment analysis to explore the possible mechanisms underlying CAVD, including GO, KEGG, DisGeNET, and TRRUST databases. Notably, the batch effect was corrected using an empirical Bayes framework in our study.

CAVD is a chronic inflammatory disease sharing similar pathogenesis with atherosclerosis [9]. The sterile inflammation in all stages of CAVD is accompanied by extensive immune cell infiltration (such as macrophages, T cells, and mast cells) [28–31]. In this process, cell adhesion molecules facilitate their adhesion and transendothelial migration via interacting with the ligands on the immune cells [32, 33]. The GO analysis revealed that leukocyte migration, extracellular structure organization, extracellular matrix organization, T cell activation, and cell-substrate adhesion were significantly enriched. These invading immune cells release pro-fibrotic and pro-inflammatory cytokines, contributing to the reactive oxygen species production, mast cell degranulation, and synthesis of valvular extracellular matrix[34], thus ultimately increasing value thickness. Among the many cytokines, TNF-α has been demonstrated as a particularly important regulator in CAVD that triggers the transition of valvular smooth muscle cells and myofibroblasts to myofibroblasts via activating Wnt pathway activation and increasing ALP, BMP-2, and RUNX2 expression [35–37]. However, the role of other pro-fibrotic and pro-inflammatory cytokines remains limited.

TNC, also named Tenascin-C, is a large extracellular matrix glycoprotein and the main component of extracellular matrix-induced dynamic mechanical stress [38]. Our study showed that *TNC* was significantly up-regulated in calcified aortic valves. TNC is transiently present during extracellular matrix remodeling and is involved in cell differentiation, proliferation, and migration [39]. In healthy valves, TNC was primarily distributed on the basement membrane beneath the endothelial cells, while stenotic valves exhibited no such distribution but immunoreactivity in the deeper layers, especially in calcific nodules [40]. Interestingly, TNC has been demonstrated to induce phenotypic changes of fibroblasts to myofibroblasts in human breast cancer. Moreover, TNC is also reported to be detrimental in cardiac fibrotic remodeling and hypertrophy after myocardial infarction, probably via up-regulating angiotensin-converting enzyme [41, 42].

In CAVD, a disorganized and disrupted valvular extracellular matrix is susceptible to the dystrophic calcification induced by calcium and phosphate deposits. Activated valve interstitial cells may differentiate into myofibroblasts and osteoblasts, contributing to the fibrosis and ossification of the aortic valve, respectively [43, 44]. SPP1 (also termed osteopontin) plays a central role in the progression of biomineralization, which accelerates reparative fibrosis and contributes to the differentiation of valvular interstitial cells into osteoblasts [45–47]. Consistent with our research, multiple studies have revealed an elevated *SPP1* expression in calcified aortic valves compared with healthy valves [48, 49]. Vadana et al. [50] analyzed the high glucose-induced calcification on gelatin-populated 3D constructs with human valvular endothelial cells and interstitial cells. The results showed increased calcium deposits and up-regulated osteogenic molecules, including SPP1, osteocalcin, and bone morphogenetic protein [50]. In previous bioinformatic research on 6 valvular tissue samples from patients with aortic stenosis, RNA-Seq analysis suggested that SPP1 was significantly up-regulated in aortic valve sclerosis [51]. Moreover, from the perspective of immune cell infiltration and cytokine response, macrophage-derived valvular extracellular matrix protein SPP1 could contribute to immune cell migration, cytokine release, as well as calcium deposition [49].

*SCG2*, or secretogranin II, is a 587 amino acid long protein from the chromogranin-secretogranin protein family [52]. We found that SCG2 was significantly up-regulated in calcific valves and was predicted to be a CAVD-related gene by WGCNA. Secretoneurin is an active neuropeptide derived from secretogranin II, an abundant protein in neuroendocrine storage vesicles [53]. Secretoneurin is a key modulator in the inflammatory process [54], including stimulating monocyte chemotaxis [55], increasing the permeability through monolayered coronary endothelial cells [56], and transendothelial transport of cells and substances [57]. Secretogranin has been demonstrated to act as a pro-angiogenic agent and induce postnatal vasculogenesis by enhancing VEGF signaling in endothelial cells [58], promoting inflammatory cell/macrophage accumulation, and stimulating VSMC proliferation [59]. As angiogenesis [60] and inflammation [8] may contribute to CAVD progression, it is necessary to ascertain the exact role of *SCG2* in CAVD.

Our study showed that *FAM20A* was significantly up-regulated in calcified aortic valves, which might contribute to valve calcification progression. The Fam20 families (including *FAM20A*, *FAM20B*, and *FAM20C*) are novel kinases phosphorylating secreted proteins and proteoglycans. FAM20A activates Fam20C, which phosphorylates hundreds of secreted proteins, whereas Fam20B could regulate proteoglycan synthesis via phosphorylating a xylose residue [61, 62]. Mutations in *FAM20A* have been demonstrated to cause amelogenesis imperfecta and enamel renal syndrome [63, 64]. However, the role of *FAM20A* in aortic valve calcification remains unclear.

CD52, also known as CAMPATH-1 antigen, is a glycoprotein of 12 amino acids anchored to glycosylphosphatidylinositol [65]. In our research, CD52 expression was significantly up-regulated in calcific valves compared with normal samples. As a nonmodulating membrane glycoprotein, it is highly expressed on the surface of immune cells (e.g., mature lymphocytes, especially memory CD4^+^ T cells and B cells, monocytes, and dendritic cells) [66]. It has been shown that CD52 may provide costimulatory signals for T-cell activation and proliferation [65]. However, Rashidi et al*.* [67] reported another formation of CD52, soluble CD52, which was associated with suppressing inflammatory cytokine production by limiting TLR suppress-induced NF-κB activation in the innate immune cell, indicating its anti-inflammatory function and immunotherapeutic agent.

Several limitations of the study should be mentioned. First, the paucity of confirmatory experiments is a significant limitation. Considering the accumulating publicly available disease-related gene expression profiles, it would be necessary to perform a comprehensive analysis and make full use of the existing resources economically and effectively. However, a further mechanism investigation based on the current dataset would be unreliable. Therefore, this study is descriptive without mechanism research, and further molecular biology experiments would be necessary to validate the results of the present study. Second, all samples were derived from male individuals. Given the sex-dependent features of CAVD [68], the sex-unbalanced samples are likely to result in selection bias. Third, although a total of 47 samples were used for analysis, the input data might still be insufficient to identify and validate key genes in the CAVD development.

## Conclusions

This study suggested that *SPP1*, *TNC*, *SCG2*, *FAM20A*, and *CD52* might be key genes associated with the development and progression of CAVD. Further studies are required to elucidate the underlying mechanisms and provide possible therapeutic targets.

## Supplementary Information

**Additional file 1.** 55 overlapped genes and their corresponding module color.

## Data Availability

All the data were acquired from the Gene Expression Omnibus (GEO) database.

## Abbreviations

CAVD: Calcific aortic valve disease
WGCNA: Weighted gene co-expression network analysis
DEG: Differentially expressed gene
GO: Gene ontology
KEGG: Kyoto Encyclopedia of Genes and Genomes
PPI: Protein-protein interaction
ME: Module eigengene
MS: Module significance
GS: Gene significance
PCA: Principal component analysis
