# Supplementary material for: Identification of key genes in calcific aortic valve disease via weighted gene co-expression network analysis
Source: BMC Med Genomics. 2021 May 21;14:135. doi: 10.1186/s12920-021-00989-w (PMC8138987; doi:10.1186/s12920-021-00989-w)
Supplement: Supplementary file 1 — Additional file 1. 55 overlapped genes and their corresponding module color. [file 12920_2021_989_MOESM1_ESM.docx]

**sTable 1. 55 overlapped genes and their corresponding module color.**

| Gene symbol | Module color |
| --- | --- |
| ACADL | yellow |
| ALDH2 | blue |
| ANXA3 | yellow |
| APCDD1L | blue |
| ATP1A2 | blue |
| ATP1B1 | yellow |
| BCAT1 | yellow |
| C6 | blue |
| CA12 | yellow |
| CCL19 | yellow |
| CD52 | blue |
| CD93 | yellow |
| COCH | yellow |
| COL6A6 | yellow |
| CTHRC1 | blue |
| EHBP1 | blue |
| ENPP2 | yellow |
| FAM20A | yellow |
| FCGR1A | yellow |
| FCGR1B | yellow |
| FREM1 | yellow |
| GMFG | yellow |
| IBSP | blue |
| IGSF10 | yellow |
| LAPTM5 | blue |
| MAOA | yellow |
| MMP12 | blue |
| MYOC | yellow |
| NDUFA4L2 | blue |
| PDZRN4 | blue |
| PLAUR | blue |
| PPAP2B | yellow |
| PTGDS | yellow |
| RAC2 | blue |
| RHOU | yellow |
| SCARA5 | yellow |
| SCG2 | blue |
| SGCE | yellow |
| SHC4 | yellow |
| SLC16A9 | yellow |
| SLPI | blue |
| SPOCK1 | blue |
| SPP1 | blue |
| STMN2 | blue |
| TCEAL2 | blue |
| THBS2 | blue |
| THY1 | blue |
| TMEM158 | yellow |
| TNC | yellow |
| TREM1 | yellow |
| TSPAN7 | yellow |
| UST | yellow |
| VAT1L | yellow |
| VMO1 | blue |
| WASF3 | yellow |
